# PACOPED QL: Development and evaluation of the quality-of-life scale for children with life-threatening illnesses

**DOI:** 10.1017/S1478951524001779

**Published:** 2025-01-13

**Authors:** Laia Riera-Negre, Javier Varona, Maria Rosa Rosselló, Sebastià Verger

**Affiliations:** 1 Department of Applied Pedagogy and Educational Psychology, Facultad de Educación. Universitat de les Illes Balears, Palma, Spain; 2 Department of Mathematics and Computer Sciences, Universitat de les Illes Balears, Palma, Spain

**Keywords:** Quality of life, pediatric palliative care, PACOPED QL, reliability, validity

## Abstract

**Objectives:**

This study aims to validate the Palliative and Complex Chronic Pediatric Patients QoL Inventory (PACOPED QL), a new quality-of-life (QoL) assessment tool for pediatric palliative patients with complex chronic conditions. The goal is to create a comprehensive and inclusive instrument tailored to this unique population, addressing the gap in existing tools that do not meet these specific needs.

**Methods:**

The validation process included a literature review and consultations with experts. A pilot study refined the items, followed by a cross-sectional study involving pediatric palliative patients and their caregivers. Statistical analyses, such as Cronbach’s alpha for internal consistency and exploratory factor analysis for structural validity, were utilized.

**Results:**

The PACOPED QL, comprising 50 items across 8 domains and 6 subdomains, demonstrated strong reliability with Cronbach’s alpha and Guttman split-half reliability both exceeding .9. Validity assessments confirmed its suitability for children with complex illnesses. The tool was refined through expert consultations and pilot testing, reducing items from an initial 85 to a final 50, ensuring relevance and clarity.

**Significance of results:**

The PACOPED QL shows strong reliability and validity in assessing QoL in pediatric palliative patients. Its comprehensive structure makes it a promising tool for clinical practice and research, addressing a critical need for a tailored assessment in this population. The instrument’s robust psychometric properties indicate its potential utility in improving the QoL assessment and care for children with life-threatening illnesses. Further studies are encouraged to confirm its effectiveness across various settings.

## Introduction

The concept of quality of life (QoL) is of special interest for practical applications and research in education and special education fields. QoL includes both subjective and objective components that can be measured, and is understood today through a multidimensional approach encompassing 8 dimensions: emotional well-being, interpersonal relationships, material well-being, personal development, physical well-being, self-determination, inclusion, and rights. These areas collectively represent the complete QoL construct. This conceptual and measurement framework of QoL has been cross-culturally validated by Schalock and Verdugo (2012a) and Schalock et al. (2002) among other authors (Gómez et al. 2010; Schalock et al. 2010, 2005; Wang et al. 2010).

Based on Schalock and Verdugo’s (2007) theoretical model of QoL, designed specifically for individuals with disabilities, the Palliative and Complex Chronic Pediatric Patients QoL Inventory (PACOPED QL) is introduced. This decision is influenced by studies indicating that between 40% and 48% of pediatric palliative patients experience cognitive impairment (Bogetz and Lemmon 2021; Feudtner et al. 2011). Schalock and Verdugo’s (2007) model is particularly suitable due to its holistic and multidimensional nature, encompassing personal autonomy, social and emotional well-being, and personal competence (Martín 2006; Verdugo et al. 2013). Adapting this model to the specific needs of the target population involves a collaborative approach with experts in pediatric palliative care, developmental psychology, and cognitive disabilities, as well as caregivers and health professionals. This multidisciplinary approach allows for the development of elements that accurately reflect the unique experiences and needs of pediatric palliative patients and their caregivers, facilitating a comprehensive assessment of their QoL.

Despite the existence of tools such as the Pediatric QoL Inventory (PedsQL) and the Child Health Questionnaire (CHQ), which have been widely used to assess QoL in pediatric populations, a latent need is identified to develop a more specific and adapted questionnaire, like the PACOPED QL, for complex chronic and palliative pediatric patients. The justification for the creation of this new instrument is supported in several critical areas that directly affect the lives of these patients and go beyond the purely health dimension. First, the inclusion of education as a key dimension reflects the growing understanding that the QoL of children with chronic or terminal conditions cannot be detached from their educational and learning environment. Adaptive educational environments and pedagogical support are crucial for these children, whose needs go beyond medical care and require a holistic approach that includes their cognitive, social, and emotional development (Bai et al. 2017; Hall et al. 2019; Paz-Lourido et al. 2020). Furthermore, the importance of social interaction and emotional well-being is recognized, aspects that can be deeply affected in children with complex health conditions (Bravo et al. 2020; Mattson et al. 2019). Inclusion and self-determination, key concepts in the model of Schalock and Verdugo (2007), highlight the need to ensure that these children not only receive appropriate medical care but are also offered the opportunity to actively participate in their community and make decisions about their lives as much as possible (Azar et al. 2020). The creation of the PACOPED QL also responds to the need for an instrument that contemplates the particularities of pediatric palliative care, which focuses not only on alleviating physical suffering but also on addressing the emotional, social, and spiritual needs of the child and their family. This approach requires a deeper and multidimensional assessment of QoL, capable of capturing the complexities inherent in caring for children with serious or terminal illnesses (Avoine-Blondin et al. 2018).

Finally, the dual design of the PACOPED QL, which allows the participation of both the patients and their main caregivers, addresses an important gap in the assessment of QoL in this population. Recognizing that the cognitive ability of these children may be compromised, the instrument ensures that their voice and experience are reflected through the perceptions of their caregivers, while allowing for the direct participation of the child whenever possible.

Taking into account all the above, the PACOPED QL instrument has been designed to specifically address the needs of complex chronic and palliative pediatric patients. The aim of the study is to validate this instrument following a comprehensive measurement model, ensuring that it effectively captures the multidimensional aspects of QoL, including physical, emotional, social aspects, among others. This approach intends to provide a reliable and sensitive tool for assessing QoL in this unique group of patients.

## Methods

For the validation of the PACOPED QL, a pilot study was carried out as a fundamental part of the methodological process. The main objectives of this pilot study were to refine the items of the instrument and to ensure the feasibility of the main study. The pilot study was conducted in 2 main phases. In the first phase, 85 items were developed based on the Schalock and Verdugo’s (2007) model and reviewed by a panel of experts in pediatric palliative care, developmental psychology, and cognitive disabilities, as well as caregivers and healthcare professionals. In the second phase, the Delphi method was used to achieve consensus on the relevance and clarity of each item through several rounds of feedback from a panel of experts (*n* = 14). Subsequently, Aiken’s *V* index was employed to assess content validity, retaining only items with high levels of agreement (*V* > .75). This process reduced the number of items from 85 to 58, and finally to 50, by eliminating those with low content validity or redundancy. These phases and procedures used in the pilot study are detailed below:

### Design

**Figure 1.**
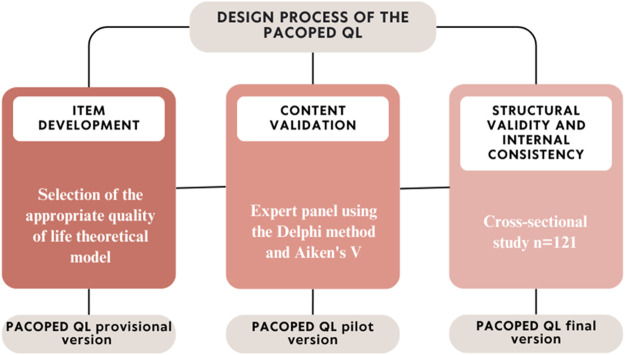
Iterative development followed in the design and validation of the PACOPED QL inventory. Based on the model proposed by Skogestad et al. (2023).

The methodology employed in this study represents a meticulous and ethical effort in developing a reliable and valid instrument to assess the QoL in pediatric palliative and complex chronic patients. This process has been carried out using an iterative model of psychometric instrument validation, based on the proposal by Kim (2009), which has allowed us to continuously refine our instrument at each stage of its development. In order to follow the principles of Open Science, the protocol has been registered on Open Science Framework (OSF) (osf.io/x7q6v). Furthermore, the project was developed considering the principles of Responsible Research and Innovation and the European Code of Conduct for Research Integrity (All European Academies 2017) and was conducted following the Declaration of Helsinki, thereby ensuring that our research practices are not only scientifically rigorous but also socially responsible and aligned with the highest European ethical standards. In addressing a highly vulnerable population, we have followed stringent ethical standards in data collection and management, ensuring the complete anonymization of the data and the protection of our participants. All participants provided written informed consent. The methodology has been evaluated and approved by the ethics committees of the University of the Balearic Islands (Comité d’Ètica de la Recerca, CER) and the University of the Americas (Comité de Ética para la Investigación en Seres Humanos). We detail below each phase of this process (see Figure 1).

### Item development

We designed a dual instrument that allows for responses from both the patient (if their cognitive abilities are intact) and the primary caregiver, or solely from the caregiver if the patient is unable. This methodology ensures the inclusion of all patients, regardless of cognitive capacity, and enhances the reliability of the instrument by maintaining question consistency, always focusing on the pediatric patient’s experience. Schalock and Verdugo’s model is particularly suitable due to its holistic and multidimensional nature, encompassing personal autonomy, social and emotional well-being, and personal competence (Martín 2006; Verdugo et al. 2013). By adapting this model to the specific needs of our target population, we engaged in a collaborative approach with experts in pediatric palliative care, developmental psychology, and cognitive disabilities, as well as caregivers and health-care professionals. This multidisciplinary approach allowed us to develop items that accurately reflect the unique experiences and needs of pediatric palliative patients and their caregivers, facilitating a comprehensive assessment of their QoL.

For the items scoring, all indicators were reflected against a 4-point Likert scale that asks about the frequency of occurrence in the last month (4 “Always,” 3 “often,” 2 “sometimes,” 1 “never”). Negative items were recoded as 5 minus the original score (Liu et al. 2022).

### Content validation

In the content validation of the instrument, an iterative process was employed to ensure optimal content consistency. Initially, the Delphi method was utilized, involving methodological experts (*n* = 2), education professionals (*n* = 7), and health-care professionals (*n* = 5). This approach allowed for the progressive refinement of the instrument’s items through expert consensus. Subsequently, the pertinence and clarity of each question were evaluated using Aiken’s *V*, following methodologies established by Roebianto et al. (2023) and Aiken (1980). This analysis provided an objective measure of content validity. Adjustments and refinements to the questions were based on this feedback, continuing the process until a high degree of saturation in content consistency was reached, at which point no further significant changes were required.

### Reliability analysis

For internal consistency, scale reliability was calculated using Cronbach’s alpha (Cronbach 1951), and stability was assessed using Spearman correlations (Hauke and Kossowski 2011). In addition, the Mann–Whitney *U* test was conducted to examine the consistency and reliability of responses between primary caregivers and patients. Continuing with our analysis of reliability, we performed a split-half reliability assessment employing Guttman’s coefficient (Chen et al. 2023). Before proceeding with the factorial analysis, the Kaiser–Meyer–Olkin (KMO) test and Bartlett’s Test of Sphericity were conducted to ensure that the dataset was suitable for factorial analysis (Seifert et al. 2019). The structural analysis involved an exploratory factor analysis (EFA) (Fabrigar et al. 1999) with a factor loading matrix, a heatmap (Skogestad et al. 2023) and a scree plot.

### Participants

The multicentric cross-sectional study involved *n* = 121 participants, including *n* = 40 pediatric palliative and/or complex chronic patients and *n* = 81 of their primary caregivers. We adopted a comprehensive approach to assess structural validity and internal consistency. The sample was heterogeneous, encompassing various disease typologies to ensure ecological validity and representativeness. Inclusion criteria were (1) pediatric patients diagnosed with complex chronic conditions and in palliative care, (2) ability to understand and respond to the questionnaire items or have a caregiver willing to participate, and (3) informed consent provided by the caregivers and, where possible, by the patients. Participants were recruited from pediatric palliative care units at HUSE in Spain and SOLCA in Ecuador. They completed an online questionnaire that included sociodemographic information and the pilot version of the PACOPED QL between November 2022 and March 2023.

## Results

### Content validity

In the validation process of the instrument, the panel of experts initially generated a set of 85 items, thereby creating the provisional version. These items were structured into 8 distinct dimensions, reflecting the fundamental aspects of QoL according to the selected theoretical model for this research. These dimensions encompass a broad spectrum of experiences and conditions relevant to the target population. Subsequently, the panel focused its efforts on refining and selecting the most appropriate items. This selection was based on criteria of relevance, comprehensibility, and discriminative ability. The intention was to ensure that each item accurately and directly reflected the various aspects of QoL in the pediatric palliative context. Through this rigorous process, the best-worded and most pertinent items were identified.

Using the Delphi method, iterative rounds of evaluation and review were conducted. This approach allowed for effective collaboration and progressive consensus among the experts. As a result of this process, the number of items was reduced to 58 (see Table 1). These items were structured on a 4-point Likert scale, including the options: “Never,” “Sometimes,” “Often,” and “Always.” A conscious decision was made to exclude a middle option to avoid the central tendency bias, which could compromise the precision of the responses. To evaluate the consistency and content validity of the selected items, Aiken’s *V* was applied. This method provided a quantitative measure of the agreement among evaluators regarding the pertinence and clarity of each item. The experts rated each item in terms of its relevance, clarity, and representativeness within the proposed scale. Items that reached a predetermined level of agreement, reflected by high values in Aiken’s *V*, were retained in the final version of the instrument [.75 – 1] (Penfield and Giacobbi 2004). This procedure ensured that each item of the PACOPED QL pilot version was both representative of the measured construct and clear and comprehensible to the study participants.

**Table 1.** Dimensions, subdimensions, and number of items per dimension in the instrument before statistical validation

	Dimension	Subdimension	Number of items
1	Physical well-being	Health indicator	3
		Physical indicator	8
		Leisure and free time indicator	3
		Social-educational-health-care indicator	3
2	Emotional well-being	Emotional indicator	4
		Concerns and fears	5
		Indicator of self-concept and self-esteem	5
3	Interpersonal relations		3
4	Social inclusion		7
5	Personal development		7
6	Material well-being		3
7	Self-determination		3
8	Rights		4
			**Total 58**

### Reliability analysis

The current section provides a detailed account of the results obtained from the validation process of an assessment tool designed to measure specific variables across 2 population groups: underage patients and their caregivers (see Table 2). Initially, an evaluation of the normality of the distributions was conducted, an essential step which informed the subsequent choice of statistical tests utilized in the assessment. The Shapiro–Wilk normality test was employed, yielding a *p* < .05, thereby warranting the use of non-parametric statistics for further analysis. Cronbach’s alpha values were calculated using Spearman’s correlations, and for both groups, these values indicate high internal consistency, suggesting that the items of the instrument coherently measure an underlying construct within this group. The internal consistency analysis for each of the items individually in both groups revealed very favorable values; hence, it is not recommended to eliminate any items from the instrument.

**Table 2.** Statistical analysis of reliability focused on the specific group and the entirety of the instrument

	Group		
Statistics	Patients/minors	Primary caregivers/adults	Instrument
Shapiro–Wilk normality test	*W* = .97222, *p* < 2.2e−16	*W* = .9841, *p* < 2.2e−16	*W* = .96554, *p* < 2.2e−16
Cronbach’s alpha internal consistency	*α* = .96	*α* = .91	*α* = .93 *α*_1_ = .83 *α*_2_ = .76 *α*_3_ = .61 *α*_4_ = .75 *α*_5_ = .8 *α*_6_ = .02 *α*_7_ = .75 *α*_8_ = .65
Guttman’s coefficient (GC)			GC = .97

Furthermore, the internal consistency of each dimension is assessed to determine the homogeneity of its items. This is done by assigning a subscript to each Cronbach’s alpha value that corresponds to the dimension number. In this way, it is ensured that each set of items within a specific dimension consistently measures the same construct, thereby contributing to the overall reliability of the instrument. In the first dimension, a satisfactory alpha value is recorded. A differential trend in responses is identified for items 15–17, associated with the Social-Educational-Health Care indicator. However, the exclusion of these items does not significantly impact the alpha value of the dimension. A similar phenomenon is observed in the fourth dimension with item 36. In contrast, the sixth dimension, consisting of 3 items, shows a notably low alpha value. Regarding the remaining dimensions, adequate internal consistency is observed. The split-half Guttman’s coefficient of the total scale is .97 which further confirms the internal consistency.

**Figure 2.**
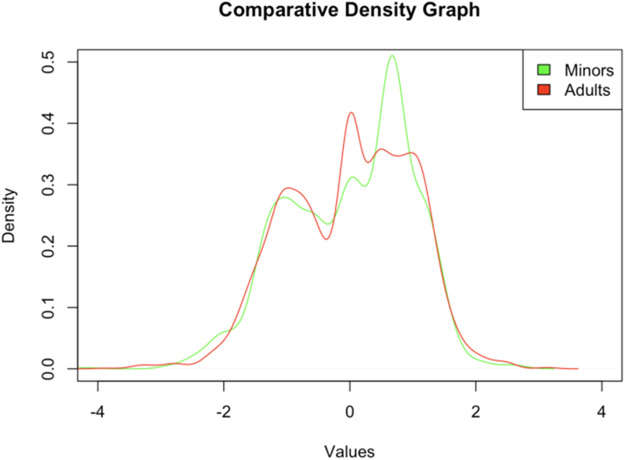
Comparative distribution of scaled responses in QoL assessment between minors and adults.

The comparative density graph (refer to Figure 2) displays the scaled distributions for 2 distinct cohorts: minors and adults, as they respond to a QoL measurement instrument. Both cohorts’ distributions exhibit a bimodal nature, with 2 predominant peaks within each group. This bimodality may indicate the presence of differentiated subgroups or the instrument’s capture of multiple underlying constructs. The density curves for both groups significantly overlap, suggesting similarities in QoL responses across the groups. However, notable differences exist in the prominence and breadth of the peaks between the 2 distributions. Specifically, the second peak in the minors’ distribution is higher and narrower compared to the corresponding peak in the adults’ distribution, implying greater uniformity in responses within this subgroup. A detailed analysis of the distributions reveals response variations that are critical for interpreting the measurement instrument’s validity. The consistency observed within the peaks may inform the internal coherence of the scales associated with the instrument’s items. The presence of variations between the responses of minors and adults suggests that the instrument is sensitive to potentially substantial differences in QoL perception across these age-groups. In light of these findings, it is observed that the instrument differentiates between different states or dimensions of QoL, as evidenced by the bimodality present in the responses of both cohorts. This characteristic of the density distributions reflects the instrument’s ability to capture the complexity and multifaceted nature of the measured construct, which is a crucial aspect in the assessment of QoL across diverse populations.

The Wilcoxon–Mann–Whitney test statistic indicates that there is insufficient statistical evidence to reject the null hypothesis of equal medians between the 2 compared groups (see Table 3). Within the context of the QoL analysis, this suggests that according to the Wilcoxon test, there is no significant difference in the median QoL scores between minors and adults within the studied sample. The alternative hypothesis, which posits that the true location difference is not equal to zero, is not supported by these data. This is in line with the visual inspection of the density plots, where the distributions demonstrated considerable overlap.

**Table 3.** Statistical analysis of the instrument’s structure

Statistics	Values
Wilcoxon–Mann–Whitney test	*W* = 5,418,795, *p* = .6988 Alternative hypothesis: true location shift is not equal to 0
Bartlett’s Test of Ssphericity	*X*^2^ = 3512.268, *p* < 2.2e−16, *df* = 1653 *p*_1_ < .001 *p*_2_ < .001 *p*_3_ < .001 *p*_4_ < .001 *p*_5_ < .001 *p*_6_ = .33 *p*_7_ < .001 *p*_8_ < .001
Kaiser–Meyer–Olkin (KMO) factor adequacy	KMO = .7 KMO_1_ = .76 KMO_2_ = .74 KMO_3_ = .56 KMO_4_ = .81 KMO_5_ = .74 KMO_6_ = .48 KMO_7_ = .63 KMO_8_ = .64

### Structural validity

**Figure 3.**
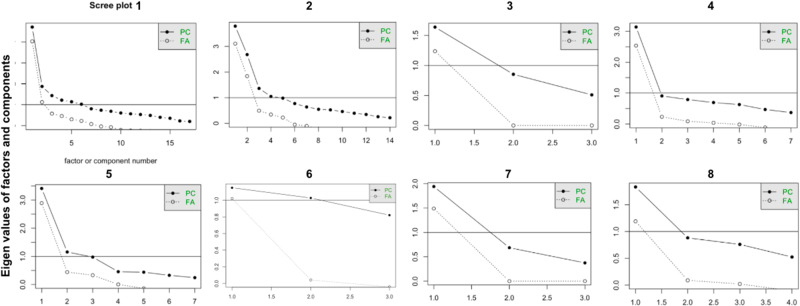
Scree plots generated from the exploratory factor analysis of each dimension.

**Figure 4.**
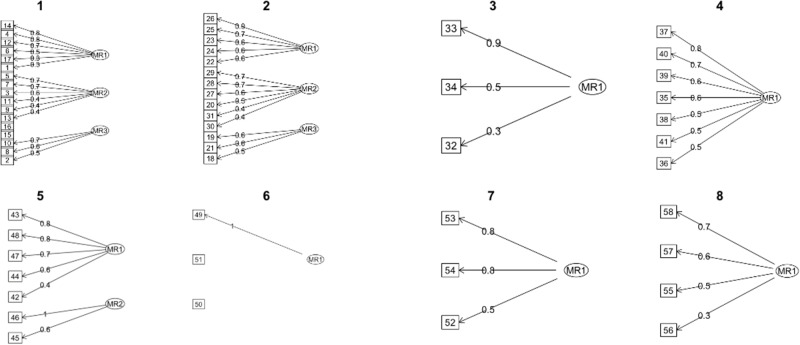
Factor analysis plots of each dimension.

For a more in-depth analysis, a dissection of each dimension is performed. To accomplish this, Bartlett’s Test of Sphericity and the KMO factor adequacy tests are calculated for the whole instrument and recalculated for each dimension (see Table 3). The aim is to confirm whether the variables are correlated in the population from which the sample was drawn, thereby justifying the utilization of dimension reduction techniques such as factor analysis (FA). Furthermore, it is examined whether the study of the instrument’s structure through FA should be considered in conjunction with theoretical interpretability (Bartlett *p* < .05; KMO > .5). Consequently, a Varimax rotation is conducted for the FA within each dimension (See Figure 3), as well as a principal component analysis, with each set of results graphically represented in a Scree Plot (see Figure 4).

The results of Bartlett’s Test of Sphericity and the KMO index are significant across all dimensions, except for dimension 6, supporting the use of FA. The root mean square residual is within the optimal range of [0; 0.1] for other dimensions, indicating adequate derived factors. This is further corroborated by the Scree plot graphs for each dimension, except dimension 6. Specifically, in dimension 2, an inverse correlation is found between questions on fears and end-of-life (items 22–26) and other items. Additionally, item 20 shifts from “Emotional Indicator” to “Self-Concept and Self-Esteem.” Issues in dimension 6 are due to response polarization, making its items irrelevant or noninformative.

In the FA process, significant results were obtained regarding the eigenvalues of the identified factors. Eigenvalues represent the variance explained by each factor, indicating their importance in the model. In our study, all 8 factors (MR1-MR8) had eigenvalues exceeding the critical value of 1, as per Kaiser’s criterion, which suggests retaining factors that explain significant variance. The SS loadings ranged from 1.451 to 5.962, with MR1 being the most dominant. The lowest eigenvalue (MR7 with 1.451) still justified retention. These results support retaining all 8 factors, providing a solid foundation for further research applications.

After conducting the reliability calculations and structural analyses, the instrument has been reduced from its original 58 items to a final set of 50 items (see Table 4).

**Table 4.** Final structure of the instrument

	Dimension	Subdimension	Number of items
1	Physical well-being	Health indicator	3
		Physical indicator	8
		Leisure and free time indicator	3
2	Emotional well-being	Emotional indicator	4
		Concerns and fears	5
		Indicator of self-concept and self-esteem	5
3	Interpersonal relations		3
4	Social inclusion		6
5	Personal development		7
6	Material well-being		1
7	Self-determination		1
8	Rights		4
			**Total 50**

## Discussion

This study aimed to design and validate the first multidimensional, transcultural QoL questionnaire for palliative and complex chronic pediatric patients in Spanish-speaking countries. Ethically and culturally, we ensured the instrument was sensitive to the patients’ and their families’ cultural needs and contexts, enhancing its relevance and applicability across various settings. Following a rigorous development process, the results indicate a reliable and valid instrument. Employing Kim’s (2009) model-based methodology allowed for continuous refinement at each development stage, adhering to high ethical and scientific standards. Through iterative content validation and reliability analysis, an instrument reflecting this vulnerable group’s unique experiences and needs was developed.

The results of the study demonstrate that the PACOPED QL shows high internal consistency (*α* > .9) and a solid factor structure, which aligns with the principles of the QoL model by Schalock and Verdugo (2007) that served as the basis for the instrument’s construction. This multidimensional model has been previously validated in populations with disabilities and is particularly suitable for assessing QoL in children with complex conditions, as highlighted by Verdugo et al. (2013). The structure identified in the EFA of the PACOPED QL, with 8 key dimensions, also aligns with the findings of Gómez et al. (2010), who emphasize the importance of capturing not only physical aspects but also emotional, social, and inclusion factors in QoL assessments. The psychometric robustness of the instrument underscores its capacity to accurately measure the experiences of pediatric patients and their caregivers, supporting the instrument’s validity and its practical application in clinical settings. Additionally, the high reliability values obtained reflect the importance of a careful cultural adaptation of the instrument, as suggested in previous studies on cross-cultural adaptation (Schalock et al. 2010; Wang et al. 2010). These results strengthen the utility of the PACOPED QL not only as an assessment tool but also as a valuable resource to guide personalized interventions for children with chronic and palliative conditions, addressing the specific needs identified by studies such as Azar et al. (2020), which emphasize the need for a holistic approach in pediatric care.

Content validation initially reduced the items from 85 to 58, focusing on key QoL aspects in pediatric palliative care. Aiken’s *V* confirmed the relevance and clarity, with high consensus among experts. Items were further reduced to 50, divided into 8 factors. Based on parsimony, 8 items were removed to simplify the model while retaining reliability. In the first dimension, Cronbach’s alpha and FA led to relocating Social-educational-health care indicators to demographics. For the fourth dimension, question 36 was moved to demographics due to polarized responses. In the sixth dimension, only item 49 was retained due to low reliability (*α* = .2) and insignificant Bartlett’s and KMO values. In the seventh dimension, only item 54 was kept for effectively covering relevant aspects.

The instrument’s internal consistency is evidenced by a high Guttman’s coefficient (.97), indicating exceptional reliability. This suggests that response variations stem from actual differences in characteristics or behaviors rather than design flaws. Such reliability is crucial for precise measurements in clinical research, psychological evaluations, and decision-making. However, it’s important to evaluate other psychometric properties, including validity, to confirm the instrument’s overall integrity and applicability.

Compared to established instruments like PedsQL and CHQ, our instrument offers a broader, nuanced view tailored to our specific audience. It adapts to patients with various cognitive abilities, involving both patients and caregivers in the evaluation. The instrument addresses dimensions pertinent to our target group, enhancing comprehensiveness and alignment with their needs. Its inclusive design applies across multiple diagnoses, making it suitable for a wide range of chronic and palliative pediatric conditions. This versatility enhances its value in diverse clinical and research settings, facilitating a holistic QoL assessment.

The study is constrained by certain limitations, notably the geographical reach and the sample’s diversity, which might limit the broader applicability of our findings. To enhance the generalizability of the results, future investigations should aim to encompass a more varied and extensive sample, and consider implementing longitudinal studies to evaluate the instrument’s consistency over time. In light of this, we are initiating a second phase of data collection aimed at conducting a confirmatory test-retest statistical analysis. This endeavor is expected to not only augment the reliability of our measurements but also shed light on the long-term stability of the evaluated constructs. By addressing these limitations in subsequent research, our objective is to fortify the validity and reliability of our conclusions, thereby laying a firmer foundation for the interpretation and practical application of our findings in the pertinent academic and professional domains.

The findings support the QoL framework for pediatric patients with chronic health challenges, enriching existing research and informing health policy development. Echoing Schalock et al. (2002), they highlight the need for a comprehensive pediatric health-care approach that includes personal growth, autonomy, social participation, and children’s rights. This approach enhances life quality for young patients. Embedding QoL principles in health research and policies for children with chronic conditions meets their diverse needs. The instrument refines QoL evaluation, offering professionals a precise, child-centric tool across sectors.

The PACOPED QL enables detailed, multidimensional assessments of QoL in children with complex chronic conditions and palliative care needs. It identifies impactful areas, facilitating personalized, data-driven care plans. Integrating PACOPED QL into clinical practice enhances early identification of physical, emotional, social, and educational challenges, leading to effective interventions. The instrument promotes evidence-based practice and continuous outcome measurement, essential for healthcare improvement. Additionally, PACOPED QL data support clinical research, providing a foundation for studies optimizing pediatric palliative care. Implementing PACOPED QL ensures a holistic, patient-centered approach, alleviating suffering and promoting overall well-being.

The instrument demonstrates commendable reliability and validity, characterized by strong internal consistency and the capacity to determine the QoL among participants. These results position the instrument as an effective resource for measuring the QoL in pediatric patients with chronic and complex conditions, holding considerable promise for reliable and impactful application in this critical area.
